# Improving coverage and completion rate of isoniazid preventive therapy among eligible HIV patients using quality improvement approaches: a case study of State Hospital, Ijebu Ode, Ogun State, Nigeria

**DOI:** 10.11604/pamj.2019.34.193.19360

**Published:** 2019-12-11

**Authors:** Olabanjo Okunlola Ogunsola, Oluseye Ajayi, Omobola Ojo, Oluwatomisin Adeyeye, Yewande Akinro, Oluwabunmi Oke, Adefolake Adebare Adurogbola, Olalere Olajide

**Affiliations:** 1 APIN Public Health Initiatives, Ibadan, Oyo State, Nigeria

**Keywords:** HIV, isoniazid preventive therapy, quality improvement

## Abstract

**Introduction:**

Tuberculosis (TB) is a major killer of people living with HIV. One key strategy to reduce the incidence of tuberculosis in HIV patients is the use of Isoniazid Preventive Therapy (IPT). However, coverage of IPT among eligible HIV clients is poor. This study aims to improve IPT coverage using quality improvement approaches that help identify the root cause and improve coverage of isoniazid preventive therapy.

**Methods:**

The quality improvement (QI) project spanned over six months corresponding to three PDSA cycles. Root causes for low IPT initiation and completion in State Hospital Ijebu Ode were identified using fishbone analysis. The root causes were subjected to prioritization matrix and implementation plan was developed for the first two root causes with the highest composite matrix scores. Longitudinal data were collected over the six months period with learning session at the end of every two-month PDSA cycle. Data was analyzed using Microsoft Excel 2010 and presented in charts and tables.

**Results:**

The two most contributory factors to low IPT initiation and completion in the facility with prioritization matrix scores of 30 and 25 respectively were poor tracking system for IPT eligible clients and poor documentation of IPT commencement in the patients care cards and IPT registers. Findings showed improvement in both IPT initiation and completion with increase in initiation rate from 11% to above 50%, and increase in completion rate from 53% to 95.4%.

**Conclusion:**

The use of quality improvement approaches can improve coverage and completion rate of IPT among eligible HIV patients. Government and health programmers should support facilities to apply QI approaches to solving health service delivery.

## Introduction

TB is a leading preventive cause of death among People Living with HIV (PLHIV). According to the Global tuberculosis report (2017), an estimated 300,000 death among PLHIV were due to tuberculosis in 2017 [1]. Africa accounts for 72% of PLHIV who had tuberculosis in 2017 [1]. Antiretroviral Therapy (ART) reduces, but does not eliminate the risk of TB disease among PLHIV [2,3]. The World Health Organization recommends Isoniazid Preventive Therapy (IPT) for PLHIV as part of the TB prevention package that includes infection control and intensified TB case finding- the 3 I's [4]. Despite the WHO recommendation and the adoption of the IPT recommendation in the Nigeria national guidelines for HIV prevention, treatment and care, the TB preventive treatment coverage among PLHIV newly enrolled in care in Nigeria was 39% [1,5]. Despite the evidence-based benefits of IPT initiation among eligible PLHIV, a number of poor resource countries like Nigeria have documented low IPT coverage, citing reasons such as stock-outs of isoniazid, adherence issues, fear of developing resistance to isoniazid, pill burden and fear of side effects [6,7]. This study therefore aims to show that using quality improvement methodology can help to identify the priority root causes of poor coverage and improve TB preventive treatment coverage in poor resource setting.

## Methods

**Research setting:** State Hospital, Ijebu-Ode is one of the APIN Public Health Initiatives-supported facilities for Improved HIV service delivery under the Centers for Disease Control iCARES grant in Ogun State. It is a secondary health facility, located in the eastern senatorial district of the State. The facility serves as a referral center for provision of secondary care for most primary health facilities in the senatorial district including the three APIN Public Health Initiatives-supported PMTCT PHCs in the area. The hospital provides pediatric and adult outpatient and inpatient care, gynecology and obstetrics services, emergency care, general and specialized surgical care, and has specialized clinic for providing ART services for PLHIV. The HIV clinic provides ART services to pediatric, non-pregnant adult clients and PMTCT services to pregnant ART clients. It has different sub-units including adherence counseling unit, consulting rooms, records, phlebotomy unit, patient waiting room and data room for EMR operations. It opens every weekday and runs block appointment for cohort of patients with similar attributes. The clinic ART dispensary is situated within the main hospital pharmacy unit, about a kilometer from the ART clinic. There is also a DOT center and a GeneXpert center for diagnosis and treatment of HIV-TB co-infected patients. It has an average patient load of one hundred and ten per clinic day with a treatment current (TX_CURR) of three thousand and seven as at 31^st^arch 2019. According to FY18 annual progress report, an average of 4 out of 1000 HIV positive patients managed in the year had TB co-infection and less than one-fourth of the patients had IPT opportunity. Ogun State is one of the thirty six States in Nigeria located in South-Western region of the country. According to the 2006 National Census, the State has a population of 3,696,999 with a population projection of 5,217,716 by end of the year 2016 [8]. The prevalence of HIV in the State was highest (1.6%) among the neighboring South-Western states according to the latest HIV sero-prevalence survey [9].

**Figure 1:**
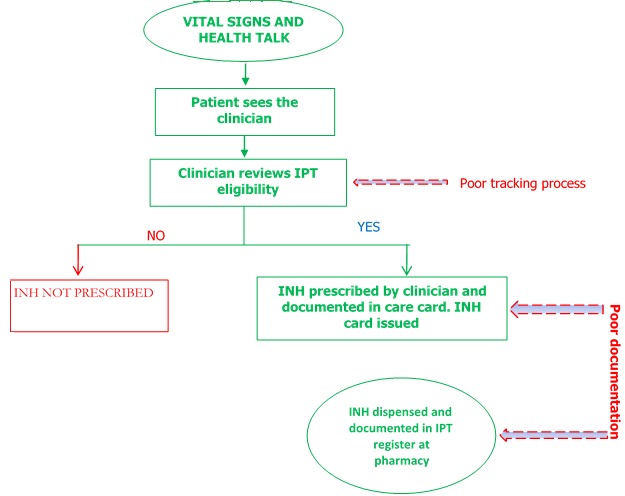
Flow of service for IPT initiation and pick up in State Hospital, Ijebu Ode

**Standard of care:** the National Guidelines for HIV Prevention, Treatment and Care recognizes Isoniazid Preventive Therapy (IPT) as the most effective approach to preventing active tuberculosis, the commonest opportunistic infection in people living with HIV (PLHIV). It prevents the progression of latent tuberculosis to active clinical disease and also increases survival rate in PLHIV. 300mg of isoniazid is administered daily as part of the comprehensive prevention package of HIV for at least six months, every two years to all adult and adolescent HIV clients with no contraindication to isoniazid use and who have had a TB screening score of zero using the WHO clinical algorithm with four cardinal symptoms; current cough, fever, weight loss and night sweat. The pediatric doses are titrated based on patient's body weight. The IPT eligibility screening in State Hospital, Ijebu-Ode is done at the ART clinic by the consulting clinicians as part of the clinic routine for all clients at every clinic contact using the national TB clinical screening algorithm in ART settings. The clinicians screen and prescribe isoniazid to the eligible clients using the pharmacy order forms and document this in the patients' care card. Thereafter, the patients are directed to the pharmacy where the drugs are dispensed to patients and efforts are captured into IPT cards, IPT register, pharmacy daily worksheet and pharmacy electronic database for record purpose (Figure 1). Prior to the quality improvement project, IPT service provision in the facility were suboptimal, with very low IPT enrollment and completion rate; this necessitated the overhauling of the service delivery processes.

**Figure 2:**
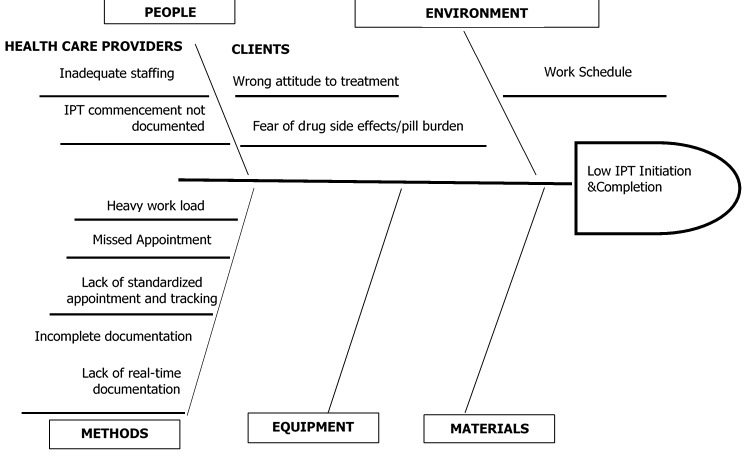
Fish bone analysis showing various root causes to IPT initiation and completion in the facility

**Project design:** a fishbone analysis was done by the IPT QI project sub-committee set up by the facility comprising an ART nurse (QI lead), an adherence counsellor, a pharmacist, a record officer and two other clinic staff. The analysis was used to itemize the root causes of the low IPT initiation and completion in the facility vis-à-vis provider-centered factors, clients-centered factors, factors related to methods, environment, equipment and commodities (Figure 2). Most contributory root causes identified were rated using prioritization matrix with each member of the committee assigning score to each root cause based on their perceived level of contribution of the factor to the problem, and ease of implementation of the remedial action. The matrix score ranges from 1 to 3 where 1 and 3 are the lowest and highest scores obtainable respectively. Implementation plan was developed for the first two root causes with the highest composite matrix scores indicating the changes to be made, strategies to effect the change, expected improvement and means of measurement i.e. indicators (Table 1). Longitudinal data were collected over a period of six months (October 2018 to March 2019) consisting of three PDSA cycles for strategies, implementation and end-of-cycle learning sessions which corresponded to the facility QI committee periodic meetings.

**Table 1:** Implementation matrix showing means of measurement

Root cause	What change will you make?	How will this change result in an improvement? i.e. How will it Work?	What improvement do we expect to see at the end of the change?	Indicators for determining improvement
1. Poor system for finding out clients eligible for IPT	- Identify clients who are eligible for IPT	- List of clients, who have not been on IPT for two years or more, is generated weekly.	- Clients who are initiated on IPT increase.	- Proportion of eligible clients’ list generated per month
	- Initiate them on IPT.	- Clients are examined; clinical records are checked for IPT eligibility.		- Number of clients who are eligible for, and commenced on, IPT.
	- Follow up till completion.			
2. Untimely documentation of IPT initiation	- Immediate documentation by clinician into the clients care card.	- Immediate documentation of IPT initiation by the clinician.	- IPT initiation will be available in the clients’ care card and IPT register.	- Number of clients initiated on IPT was documented by the clinician.
	- Immediate documentation by the pharmacist into IPT register.	- Timely documentation of IPT commencement into IPT register and pharmacy database.		- Number of clients initiated on and documented in IPT register.

**Measurements and analysis plan:** indicators measured in this quality improvement project were; Number of IPT eligible clients in a month, proportion of eligible clients who commenced IPT in a month and documented by the clinician; proportion of clients who commenced IPT in a month and documented in IPT register, proportion of clients who completed IPT among cohort that were expected to complete within the review period (October 2018-March 2019). Data were analyzed using Microsoft excel 2010 and presented in tables and charts.

## Results

**Figure 3:**
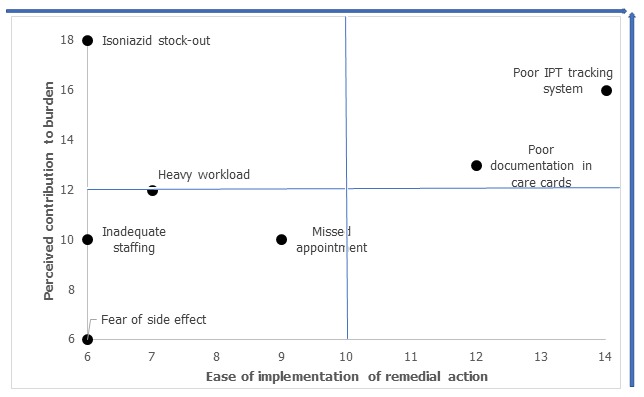
Facility IPT root causes prioritization matrix

**Figure 4:**
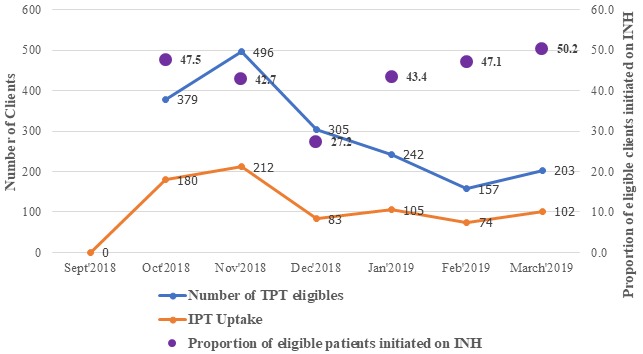
Proportion of eligible patients initiated in the facility from October 2018 to February 2019

**Figure 5:**
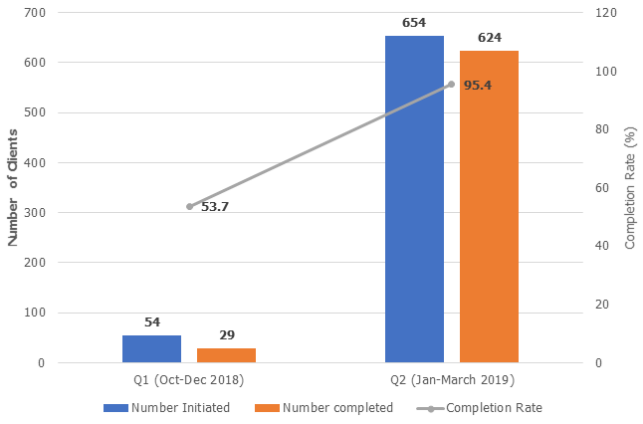
Six months IPT completion rate for cohort of patients who completed IPT in Q1 & Q2

The two most contributory factors to low IPT initiation in the facility with prioritization matrix scores of 30 and 25 respectively were poor tracking system for IPT-eligible clients and poor documentation of IPT commencement in the patients' care cards and IPT registers. Other factors considered with lower matrix scores were limited isoniazid stock/stock out, fear of side effects, inadequate staffing, missed appointment and heavy workload at the clinic (Figure 3). Findings showed a sudden rise in proportion of IPT uptake and documentation in the facility from 11% at the beginning of the project implementation to 47.5% and 42.7% in the 1st PDSA cycle in October-November 2018. There was a slight decrease in the uptake in the first month of second PDSA cycle (Dec 2018) as a result of Christmas festival; however, the trend was reversed in the late 2nd PDSA and 3rd PDSA with IPT uptake of 43.4%, 47.4% and 50.2% in January, February and March respectively (Figure 4). There was also improvement in IPT completion rate with completion rate of 95.4% among patients who were initiated between August to October 2018 and completed in January to March 2019, compared to patients who were initiated between May to July 2018 and completed in October to December 2018 (53.7%) (Figure 5).

## Discussion

In developing countries, isoniazid preventive therapy for PLHIV has shown not to have received much emphasis [10]. The 2013 WHO TB control report estimated that less than 0.5% of HIV infected persons worldwide received IPT [11]. The overview of this study is to show that the use of quality improvement methods can help to prioritize the root causes of poor coverage of IPT and also improve TB preventive treatment coverage in poor resource setting. A preventive therapy service requires supervision of clients for a long period of time which may not be effective if there is a large work load of clients by the health personnel. In this study, it was discovered that poor tracking system of patients eligible for IPT was a contributory factor to a low uptake of this medication which was similar to result found in a study with 0.4% coverage rate of IPT among eligible PLHIV [12]. This is less than the ideal coverage of a preventive service proven to reduce morbidity and to a lesser extent mortality related TB among PLHIV. However, the other matrix score with high contributory factor to poor coverage of IPT in this study was poor documentation of IPT commencement in the care cards and IPT registers. One can really deduce that there is a need to enforce role of ART treatment programs as they bring in more organization and resources through effective trainings to build capacity of the health care workers. The matrix ranking method used is a versatile tool to help the professionals facilitate decision making and determine the sequence in which to attack the problem or work towards the objective. Other factor with lower matrix scores in the study is missed appointments which was in relation to what was found in a study by Mesele Mindachew *et al.* [13].

In organizational context, the heavy workload on health care providers from initiating IPT, considering the fear of adherence and associated side effects which may occur among the patients is a key issue that must be focused on. Heavy workload among health providers can often result in compromised quality and should be addressed as part of organization context reforms to support IPT [14] which gave a clearer picture of the findings in our study. Furthermore in our findings, the main barrier hindering implementation of IPT uptake were predominantly related to Isoniazid supply problems of (stock out) concerns and the development side effects which was similar to what was found in another study in Ethiopia on PLHIV [15]. The non- availability of INH was also reported by Getahun *et al.* [11] in their study. Differences in access to Isoniazid could have contributed to the lower coverage observed in the Northern part of Teklay [15]. The side effects from a study by Durovni *et al.* [16] reported that 1.2% of persons initiating IPT discontinued therapy due to adverse reactions which is similar to factors identified as a low contributory factor of side effects in this study. The uptake rate of IPT was above the current national coverage but fell below the national set target of 90% [17] in a study in Kenya as a contrast to what was observed as low uptake of IPT in this study.

## Conclusion

Using Quality improvement approach, the facility demonstrated tremendous improvement in IPT commencement and completion over a period of six months among patients who received ART care in their HIV clinic by improving on the system of tracking eligible clients for IPT and documentation of IPT services. A notable limitation to the QI project performance with regard to IPT commencement was the limited stock of isoniazid in the country during the period of implementation. Government and health programmers are encouraged to empower facilities to contextualize their problems and apply QI approaches to improving health service delivery in the country.

### What is known about this topic

- Tuberculosis is the commonest opportunistic infection and major cause of death among people living with HIV;
- Isoniazid preventive therapy is a major strategy for tuberculosis prevention among people living with HIV;
- Coverage of Isoniazid preventive therapy is low among eligible PLHIV.

### What this study adds

- Use of quality improvement approach to identify root causes of poor IPT coverage;
- Documentation, coverage and completion of isoniazid preventive therapy can be improved using quality improvement approaches.

## Competing interests

The authors declare no competing interests.
